# Cellular Senescence in Age-Related Macular Degeneration: Can Autophagy and DNA Damage Response Play a Role?

**DOI:** 10.1155/2017/5293258

**Published:** 2017-11-01

**Authors:** Janusz Blasiak, Malgorzata Piechota, Elzbieta Pawlowska, Magdalena Szatkowska, Ewa Sikora, Kai Kaarniranta

**Affiliations:** ^1^Department of Molecular Genetics, University of Lodz, Pomorska 141/143, 90-236 Lodz, Poland; ^2^Laboratory of Molecular Basis of Behavior, Nencki Institute of Experimental Biology, Polish Academy of Sciences, Pasteura 3, 02-093 Warsaw, Poland; ^3^Department of Orthodontics, Medical University of Lodz, Pomorska 251, 92-216 Lodz, Poland; ^4^Laboratory of Molecular Bases of Aging, Nencki Institute of Experimental Biology, Polish Academy of Sciences, Pasteura 3, 02-093 Warsaw, Poland; ^5^Department of Ophthalmology, University of Eastern Finland, 70211 Kuopio, Finland; ^6^Department of Ophthalmology, Kuopio University Hospital, 70029 Kuopio, Finland

## Abstract

Age-related macular degeneration (AMD) is the main reason of blindness in developed countries. Aging is the main AMD risk factor. Oxidative stress, inflammation and some genetic factors play a role in AMD pathogenesis. AMD is associated with the degradation of retinal pigment epithelium (RPE) cells, photoreceptors, and choriocapillaris. Lost RPE cells in the central retina can be replaced by their peripheral counterparts. However, if they are senescent, degenerated regions in the macula cannot be regenerated. Oxidative stress, a main factor of AMD pathogenesis, can induce DNA damage response (DDR), autophagy, and cell senescence. Moreover, cell senescence is involved in the pathogenesis of many age-related diseases. Cell senescence is the state of permanent cellular division arrest and concerns only mitotic cells. RPE cells, although quiescent in the retina, can proliferate *in vitro*. They can also undergo oxidative stress-induced senescence. Therefore, cellular senescence can be considered as an important molecular pathway of AMD pathology, resulting in an inability of the macula to regenerate after degeneration of RPE cells caused by a factor inducing DDR and autophagy. It is too early to speculate about the role of the mutual interplay between cell senescence, autophagy, and DDR, but this subject is worth further studies.

## 1. Introduction

Age-related macular degeneration affects the macula, a specific structure in the central retina, leading to worsening of visual acuity. It is the major cause of blindness in the elderly in developed countries. Its global pooled prevalence is estimated to be more than 8%. It is an emerging problem, as it is estimated that the number of people affected by AMD in 2020 will be about 200 million, increasing to almost 300 million in 2040 [1, 2]. Medical cost of care about AMD patients is high reaching over 2 billion dollars in the USA and Australia and about a hundred million euros in some European countries [3]. Therefore, AMD is an emerging element of the global issue of vision loss and medical care.

AMD is a complex disease in which both genetic and environmental factors play a role, but the exact mechanism of its pathogenesis is unknown. The disease occurs in two forms: dry and wet. Molecular studies addressing AMD are impeded by inaccessibility of the live retina tissue from AMD patients. No effective treatment for the more common, dry form of AMD has been established yet.

**Figure 1:**
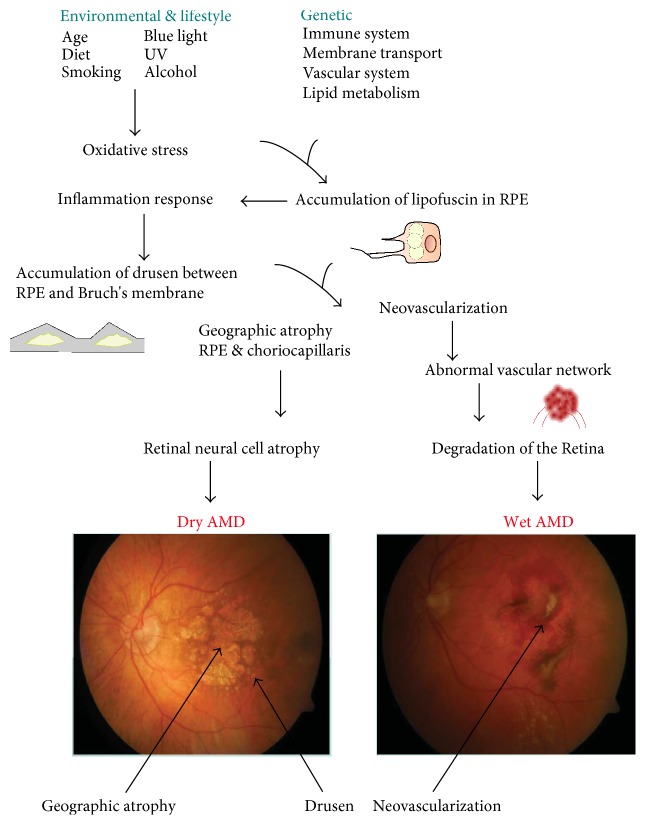
The exact mechanism of AMD pathogenesis is not known, but several factors can be implicated with a distinct role of aging. Besides aging, various oxidative stress-related environmental and lifestyle influences can be involved. The complement gene mutations play a major role in AMD. Oxidative stress and presumably other factors lead to accumulation of heterogenous lysosomal lipofuscin in retinal pigment epithelium (RPE), which induces a proinflammatory response. This, in turn, can lead to accumulation of extracellular drusen. Lipofuscin contains proangiogenic factors, such as A2E, that may develop choroidal neovascularization typical for wet AMD.

AMD affects mainly elderly people, and it is a major reason for blindness among individuals over 65 years in developed countries [4]. Aging is the most important risk factor for AMD. Although the exact mechanism of AMD pathogenesis is not known, oxidative stress, protein aggregation, and inflammation as well as some genetic factors play a central role in AMD development (Figure 1) [5]. Early dry AMD is hardly detectable and usually asymptomatic. Its advanced form, called geographic atrophy (GA), is associated with a massive loss of photoreceptors that evokes central visual loss [6]. A clinical hallmark of wet AMD is the presence of neovascular vessels sprouting from the choriocapillaris into the retina.

**Figure 2:**
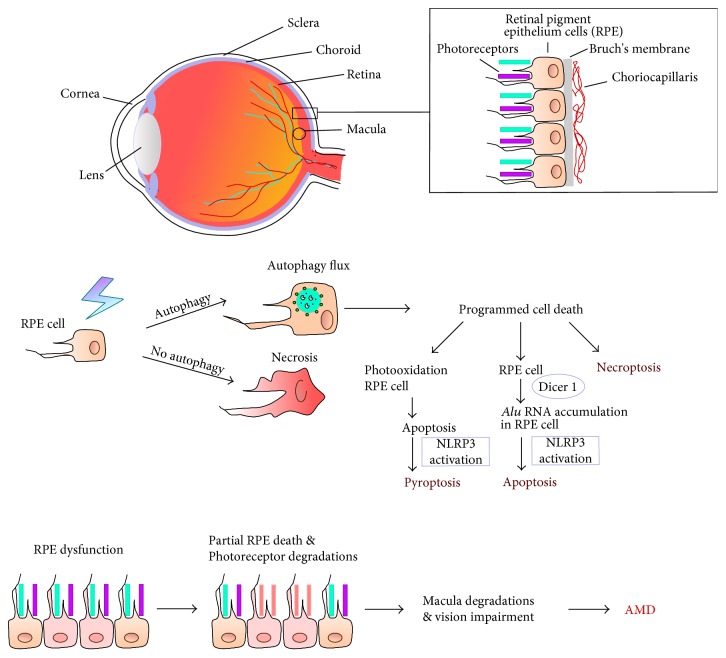
Cell death and autophagy in AMD progression. AMD affects the macula, a part in the central retina, and is associated with degradation of retinal pigment epithelium (RPE) cells, photoreceptors, and choriocapillaris. Autophagy can be decisive in switching between programmed and nonprogrammed cell death mode. Apoptosis of RPE cells can be linked to blue light exposure (photooxidation), oxidative stress, accumulation of *Alu* transposons due to impaired functioning of the DICER1 endonuclease, and the activation of the NLRP3 inflammasome. Pyroptosis can also result from photooxidation and activation of the NLRP3 inflammasome. Oxidative stress and other factors can induce necroptosis, a programmed version of necrosis.

Progression of AMD ultimately leads to RPE and photoreceptors death via several mechanisms, including apoptosis, pyroptosis, necroptosis, and necrosis [7]. Autophagy may be involved in the regulation of the cell death mode in AMD [8] (Figure 2).

## 2. Cell Senescence and Aging in AMD

Kozlowski proposed that cellular senescence of RPE cells played a role in the etiology of AMD [9]. It seems that many studies on the role of cell senescence in organismal aging and age-related pathologies support this idea.

Senescence of human fibroblasts, described for the first time by Hayflick and Moorehead [10, 11] as a cell division limit in culture, affects not only fibroblasts but also other proliferating somatic human cells, such as keratinocytes and melanocytes [12], lymphocytes [13], epithelial [14] and endothelial [15] cells, vascular smooth muscle [16, 17], mesothelial cells [18], mesenchymal stem cells [19], and even cancer cells [20, 21].

Many studies suggest involvement or even a causative role of cell senescence in aging and age-related diseases [22–25]. Indeed, using different set of markers, senescent cells were found *in vivo* in human, baboon, and mouse skin, human and rodent vascular endothelium, smooth and skeletal muscles, fat tissue and liver [26], skeletal muscle of rodents and primates [27], and human T cells [13]. There is emerging experimental evidence of the accumulation of senescent cells at sites of pathology, such as type 2 diabetes, atherosclerosis, hypertension, chronic pulmonary disease, cataracts, and glaucoma [28]. Senescent cells were also found in RPE of primates [29].

It was postulated that the exposure of cells to recurrent or chronic nonlethal stress might contribute to an increase in the accumulation of stress-induced senescent cells, thereby accelerating tissue aging [30]. Although we believe that senescent cells accumulate with age partially due to their resistance to apoptosis [31], one cannot exclude that at least some of them are cleared by the immune system, as recently reported [32] or that in certain circumstances they can die. Eradication of senescent cells by forcing them to undergo apoptosis is a subject of genetic manipulation [33, 34] or pharmacological interventions by using senolytic agents and can prolong health span [35]. On the other hand, age-dependent apoptosis of muscle cells (sarcopenia) is an undesirable hallmark of the process of organismal senescence, which can be more common than expected [36].

From a mechanistic point of view, a growing body of evidence proves that persistent DNA damage, especially double-strand breaks (DSBs) and DNA damage response (DDR), are closely associated with cell senescence [37]. Number of DSB sensor, *γ*-H2AX *foci,* a marker of DSBs, increased in both mouse and human senescent primary cells in tissue culture [38] and in the skin of old primates [39]. Senescence-associated galactosidase- (SA-*β*-gal-) positive cells and *γ*-H2AX-positive cells colocalize in old mice [40], and the number of *γ*-H2AX *foci* in lymphocytes in humans increases with age [41, 42]. Fibroblasts from individuals suffering from progeria (Hutchinson-Gilford syndrome) persistently displayed many markers of increased basal DDR [43]. Recently, it has been shown that a controlled induction of DSBs in mouse liver induces features of tissue aging [44].

**Figure 3:**
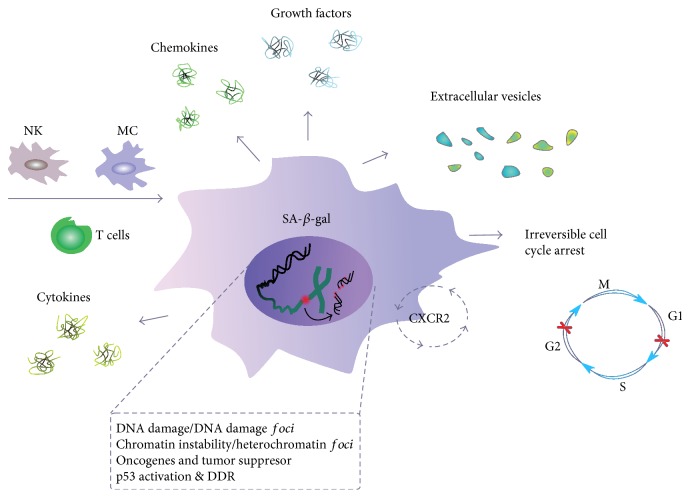
Senescent cells and senescence-associated secretory phenotype (SASP). A cell undergoing senescence is permanently arrested in the G1 or G2 phase of the cell cycle and has changed morphology. It is featured by an increased activity of senescence-associated-*β*-galactosidase (SA-*β*-gal) and can be targeted by the immunological system, with natural killer (NK) cells, macrophages (MS), and T-lymphocytes involved. Released various soluble agents, including cytokines, chemokines, growth factors, and extracellular vesicles, are main determinants of SASP. A senescent cell is characterized by an elevated level of DNA damage and chromosomes aberrations, which are also signs of genomic and chromosomal instability, typical for cancer cells. Chemokine signaling through the CXCR2 protein increases senescence.

All senescent cells display common features, such as arrest in the G1 or G2 phase of the cell cycle, increased cell size, granularity, and increased activity of SA-*β*-gal (Figure 3) [24]. Senescent cells stay alive and are metabolically active and secrete a lot of factors [45] that can be classified into inflammatory chemokines and cytokines, matrix-remodeling proteases, and growth factors [46]. SASP can not only influence tissue surveillance in a way which can promote tissue repair, prevent fibrosis, and signal to the innate immune system to clear the senescent cells but also induce cancer development and other age-related diseases [47]. It also promotes a low-grade inflammation, which can drive organismal aging (inflammaging) [48].

There is a growing body of evidence linking DNA damage with inflammation and disease, particularly age-dependent diseases [49]. This is sort of a vicious cycle as DNA damage-dependent senescence can lead to secretion of molecules, which can reinforce senescence [50] and can induce DNA damage and DNA damage-dependent bystander senescence [51].

Initiation and maintenance of the SASP requires the DDR proteins ATM, NBS1, and CHK2, but not p53 and pRb. NF-*κ*B signaling is another pathway involved in generating SASP that can be linked with DDR [52]. Recently, it has been shown that the GATA4 protein is directly involved in SASP. GATA4 is normally degraded, but it is stabilized in cells undergoing senescence. GATA4 is activated by DNA damage response regulators, ATM and ATR, but not by p53 or p16. This transcription factor activates NF-*κ*B to initiate SASP and facilitate senescence [53]. However, NF-*κ*B can be also activated by p38MAPK independently of DDR [54]. Also, mTOR can be involved in SASP as its inhibition by rapamycin substantially reduces the level of secreted cytokines [55, 56].

Although the causative role of reactive oxygen species (ROS) in aging is disputable, the paradigm assuming that oxidative stress and ROS produced by mitochondria play an important role in cell senescence has been supported [57, 58]. Hydrogen peroxide was the first factor used to show oxidative stress-induced senescence [59]. We also used this compound to show a canonical signaling pathway involved in cell senescence [60]. Oxidative stress, which can induce cellular damage, has been closely connected with the pathogenesis of AMD as the retina is particularly susceptible to the stress because of its high consumption of oxygen, high proportion of polyunsaturated fatty acids, and exposure to visible light [61]. Therefore, retinal cells can be prone to stress-induced senescence. The retina is built of three layers of neural cells and one layer of RPE cells, which are quiescent.

Although cell senescence per definition denotes a permanent growth arrest of proliferation competent cells, recently, Jurk and others have shown that some features of cell senescence, including DDR, also apply to postmitotic neurons *in vivo* [62]. However, we showed that the SA-*β*-gal phenotype in neurons could not be attributed uniquely to cell senescence either *in vitro* or *in vivo* [63].

Although epithelial cells stay quiescent in the retina, they are proliferation-prone and vulnerable to oxidative stress-induced senescence. Indeed, in several studies using proliferating human RPE-derived ARPE-19 cells, which proliferate *in vitro*, the cell senescence process was documented upon oxidative stress. In several studies, senescence was induced by hydroxyl peroxide in nontoxic concentrations [64–67]. In other studies, *tert*-butyl hydroperoxide [68] or cigarette smoke [69] was applied. Arend et al. observed a significant increase in cell viability and reduced SA-*β*-gal activity, ROS amount, and DNA damage *foci* in ARPE-19 cells induced to senescence with H_2_O_2_ and pretreated with idebenone, which is a derivative of coenzyme Q10, but with a tenfold higher antioxidant capacity than its parental compound [64]. Similarly, fullerenol, a ROS scavenger and antioxidant, protected ARPE-19 cells from H_2_O_2_-induced senescence. Interestingly, fullerenol activated SIRT1, which belongs to the family of “proteins of youth”—sirtuins [70].

The use of ARPE-19 cells in senescence studies have some limitations. ARPE-19 population can contain a substantial, if not the major, fraction of cells which are able to double their population to over 270 times, so they can be considered as immortal [23]. Unlike cells with limited number of divisions, immortal cells do not undergo replicative senescence. However, it was shown that they are prone to stress-induced senescence [71, 72].

## 3. Autophagy and DDR-Dependent or DDR-Independent Players in AMD Pathogenesis

**Figure 4:**
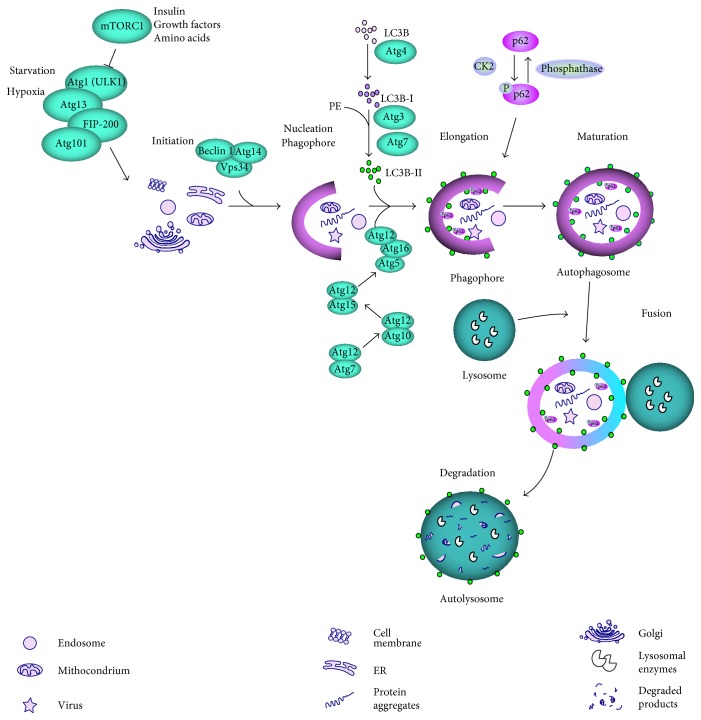
Autophagy dependent on mTOR. In normal nutrient conditions, the mTOR complex 1 (mTORC1) inhibits the ULK1 complex, consisting of ULK1, Atg13, Atg101, and FIP200, which can activate autophagy in stress conditions, including starvation and hypoxia or when the inhibitory effect of mTORC1 is abolished by growth factors, insulin, amino acids, or other agents. The material to be degraded (cargo) is then enclosed by a nucleating phagophore, which requires a translocation of ULK1 to endoplasmic reticulum (ER). ER membrane is used to form the phagophore, but other sources are also possible. The phagophore membrane is elongated, which leads to the formation of autophagosome, a vesicle with the enclosed cargo. This process is assisted by LC3 lipidated by phosphatidylethanolamine (PE) and many individual proteins, including Beclin 1, Vps34, and autophagy-related proteins (Atgs). The p62 protein functions as a selective autophagy receptor for degradation of ubiqutinated substrates, but it is itself a specific substrate for autophagy after its phosphorylation and can be selectively incorporated into the autophagosome and degraded. Fusion of autophagosome with lysosome creates autolysosome in which the cargo is degraded by lysosomal enzymes. Autophagy can be also activated by mTOR-independent pathways.

Autophagy controls cellular homeostasis by degrading in lysosomes damaged, nonfunctional or no longer needed cellular components, including organelles. Autophagic degradation provides energy, and lysosomal machinery can deliver amino acids and other degradation products back to the cytoplasm, where they can be reused as building blocks in cellular metabolism (“recycling”) [73]. This process can be carried out through at least three distinguished pathways: macroautophagy (further referred to as autophagy), chaperone-mediated autophagy (CMA), and microautophagy. Many proteins are involved in autophagy, including autophagy-related proteins (ATGs), mammalian target of rapamycin (mTOR), the serine/threonine kinase (ULK1), FIP-200, p62 (SQSTM1), and microtubule-associated protein light chain 3 (LC3) [74]. The hallmark of autophagy and its critical stage is the formation of a double-membraned vesicle enclosing materials for degradation (cargo), called the autophagosome (Figure 4). It then fuses with the lysosome forming autolysosome, in which the cargo is degraded [75].

Impaired autophagy was observed in serious human disorders, such as cancer and neurodegenerative diseases including AMD [76]. In general, autophagy plays an important role in the functioning of RPE cells [77–79]. Drusen are a clinical hallmark of AMD and an important element of its pathogenesis [5, 80, 81]. They are yellowish deposits between RPE cells and Bruch's membrane. Consequently, impaired autophagy can lead to drusen accumulation contributing to AMD development [82]. However, the relationship between autophagy and aging is not fully known and the activity of this process changes during lifetime. In addition, detailed autophagic pathways involved in the development of AMD have not been identified as different mechanisms of autophagy can function in normal and pathological retinas [83].

There is an emerging body of experimental evidence on the involvement of autophagy in AMD pathogenesis. These experiments are performed mainly on animal model of AMD and retinas obtained postmortem from AMD donors [78]. Autophagy is closely associated with cellular response to oxidative stress with the involvement of the p62/Keap1/Nrf2 pathway [84, 85]. Moreover, autophagy can be considered as an element of DDR, in which DNA repair is a major component, being a major constituent of cellular antioxidant defense [86–88]. We and others showed that AMD could be associated with disturbed DNA repair [49, 89–91].

Oxidative stress, a main factor in AMD pathogenesis, results in various DNA lesions, and 8-oxo-7,8-dihydroguanine (8-oxoG) is a hallmark of oxidative DNA damage and a major mutagenic intermediate of oxidative stress [92]. In most cases, the repair of 8-oxoG is initiated by the hOGG1 glycosylase via the base excision repair (BER) pathway. If 8-oxoG escapes this process and replicative DNA polymerase misinserts adenine instead of cytosine opposite to 8-oxoG, an alternative pathway of BER can be activated with the hMYH (MUTYH) glycosylase, which removes that adenine. Our observations revealed that genetic variability in the *hOGG1* and *hMYH* genes may be associated with AMD occurrence and progression [93]. It was reported that the level of 8-oxoG was higher in patients with exudative AMD than in control individuals. This led to the conclusion that DNA damage may underline the role of oxidative stress in AMD pathology.

Our earlier data indicated that lymphocytes isolated from AMD patients displayed a higher level of endogenous DNA damage than lymphocytes from control individuals [89]. Also, oxidative DNA damage was higher in AMD patients than in controls and cells from the patients were more sensitive to hydrogen peroxide and UV radiation, which allowed us to speculate that the combination of impaired DNA repair and elevated sensitivity to UV radiation can be important for AMD pathogenesis.

**Figure 5:**
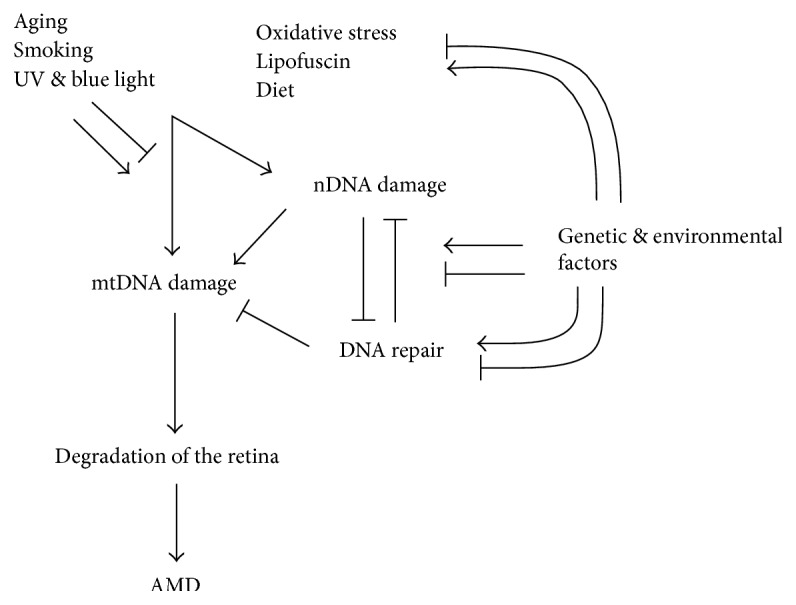
Nuclear and mitochondrial DNA (nDNA and mtDNA) can be damaged by AMD risk factors, which can also affect proteins, including DNA repair proteins. Nonrepaired or misrepaired DNA can contribute to retinal cell death occurring in AMD [89].

Not only nuclear DNA (nDNA) but also its mitochondrial counterpart (mtDNA) was reported to have elevated extent of damage in AMD (Figure 5) [90, 94–96]. Moreover, in those studies, performed on macular and peripheral RPE cells obtained from AMD patients and rodents, an increase in heteroplasmic mutations and a decrease in the efficacy of mtDNA repair were observed. In fact, there are reports showing that mtDNA in cultured RPE cells is more prone to DNA damage, at least induced by certain agents, than nDNA [91, 97–101]. Although the efficacy of DNA repair declines with age for both kinds of DNA, mtDNA from the retina was shown to have more potentially detrimental changes, than mtDNA from blood, for both normal and AMD samples [102]. These changes are potentially detrimental, if not repaired, as mtDNA contains almost exclusively coding sequences. The repair of mtDNA is generally considered as poorer than in the nucleus. All DNA repair proteins are encoded in nDNA, so its damage can affect protection from damage in mtDNA. Therefore, DNA damage can be associated with AMD occurrence and progression, suggesting that DDR can play a role in the pathogenesis of this disease.

## 4. Relationship between Senescence, Autophagy, and DNA Damage Response in RPE Cells

Many reports link oxidative stress with autophagy showing that this association is regulated in a highly coordinated pathway [103]. It is clearly illustrated by the activation of Nrf2, a transcription factor crucial for cellular antioxidant defense, by p62, a key regulator of autophagy [104]. Likely, the most direct association between oxidative stress and autophagy is expressed by mitophagy, when mitochondria with highly damaged DNA are degraded [105]. This is a specific feature of DDR in mitochondria, as in the nucleus heavily damaged DNA can induce a programmed death or in certain circumstances can be tolerated, which usually results in mutations [106]. Although ROS can induce autophagy in starvation conditions, it is not known which species are responsible for this effect [107] and both superoxide radical (O_2_^•−^) and hydrogen peroxide were considered to trigger autophagy in starvation [108–111]. In general, ROS are inducers of autophagy [112, 113]. Moreover, some data suggest that mitochondria are the main source of ROS needed for the induction of autophagy [109, 110, 114].

Several DDR pathways prevent or cope with DNA damage. However, if DNA is highly damaged, cells remain quiescent or undergo programmed cell death. Persistent, unrepaired DNA damage is typical for cell senescence. Autophagy acts as both a prosurvival mechanism and a kind of cell death, making a critical contribution to cell fate after DNA damage. Some reports suggest that autophagy delays apoptosis induced by DNA damage, providing energy required for DNA repair [1]. In general, autophagy participates in DDR by elimination of toxic aggregates, which can be a source of ROS and in this way indirectly decrease DNA damage [115, 116].

Several DDR proteins are involved in the regulation of autophagy. PolyADP-ribose polymerase 1 (PARP1), which is essential for DNA single-strand break repair, catalyzes polyribosylation of nuclear proteins converting NAD^+^ into polymers of polyADP-ribose. This leads to NAD^+^ utilization and ATP depletion, resulting in an energetic imbalance, which activates autophagy via the AMPK pathway, to recycle metabolic precursors for ATP and provide energy needed for DDR [117]. ATM, a crucial protein for DDR signaling, is another protein linking DDR to autophagy. It can activate TSC2, a tumor suppressor, to inhibit mTORC1 and induce autophagy [118]. The p53 protein, a key DDR regulator, and members of its family were shown to affect expression of several genes encoding autophagic proteins [119, 120].

It is generally accepted that autophagy declines with aging of model organism, resulting in the accumulation of cellular debris and turning the cell brown. Decreased autophagy is often associated with accelerated aging, whereas stimulated autophagy can exert a potent antiaging effect [121]. It is associated with reduced expression of proteins important for autophagy induction, including ATGs, Sirtuin 1, Beclin 1, ULK1, and LC3 [122].

DNA damage, with the key proteins ATM and ATR is a causative signal to cellular senescence. DNA damage-dependent senescence was shown in human and murine fibroblasts upon different stimuli [37]. The role of mitochondrial homeostasis and ROS generation in the process of aging has been subject of debate. Free-radical and mitochondrial theories of aging speculate that cumulative damage to mitochondria and mitochondrial DNA induced by ROS is one of the causes of aging. Oxidative damage affects replication and transcription of mtDNA and results in a decline in mitochondrial functions, which in turn leads to enhanced ROS production and further damage to mtDNA [58]. However, we showed that a decreased ROS level did not protect cells against senescence [21, 123]. Correia-Melo et al. observed that senescent cells had an increased mitochondrial mass driven by mitochondrial biogenesis, which resulted in increased cellular oxygen consumption. They have also uncovered a novel senescence regulatory pathway, in which the activation of the ATM, AKT, and mTOR phosphorylation cascades downstream of DNA damage triggered PGC-1*α*- (peroxisome proliferator-activated receptor-gamma coactivator 1 alpha-) dependent mitochondrial biogenesis [57]. However, other recent studies have highlighted mTOR as a SASP regulator by alternative mechanisms emphasizing mTOR rather as an antisenescence target [55]. Therefore, the role of mitochondrial biogenesis, ROS, and mTOR in cell senescence and SASP is still an open question.

The interplay between cell senescence and autophagy is yet unclear. Young et al. have shown that autophagy is activated upon an induction of cell senescence and contributes to the establishment of senescence [124], but there are contradictory data showing that inhibition of autophagy can favor cell senescence and that autophagy is necessary for senescence [21, 123]. We showed an impaired autophagy in RPE upon chronic oxidative stress, but senescence was not induced in that study [125]. Senescence of RPE cells may be associated with alterations in PGC-1*α* function. In neurons as well as in RPE cells, PGC-1*α* was shown to regulate lysosomal activity by TFEB protein, which might be important for improvement of autophagy flux and removal of cell damage [126, 127]. It was also demonstrated in that work that PGC-1*α* deficient mice developed some abnormalities in RPE, which were associated with their accelerated senescence. Next, PGC-1*α* alpha silencing in ARPE-19 cells aggravated H_2_O_2_-induced senescence. These cells displayed a significantly higher SA-*β*-gal activity than control cells. Senescence of RPE cells has been associated with an altered mTOR signaling [128, 129].

**Figure 6:**
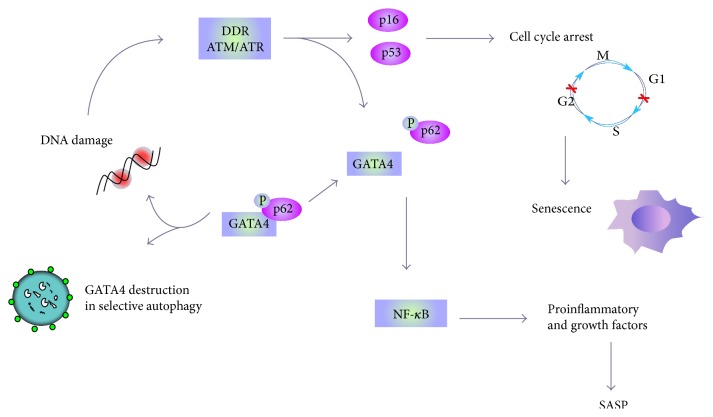
GATA4 can be involved in autophagy, senescence, and DNA damage response (DDR). The level of GATA4 is normally regulated by p62-dependent selective autophagy, but DNA damage and resulting DDR can release GATA4 from p62 control by its ATM-induced phosphorylation. If DNA damage cannot be repaired, DDR effectors induce permanent and irreversible cell cycle arrest, which is a hallmark of senescence with senescence-associated phenotype (SASP). GATA4 released from autophagic degradation can transactivate several genes that activate NF-*κ*B, resulting in the release of growth factors, chemokines, cytokines, and other molecules typical for SASP.

GATA4 is a member of GATA transcription factors, and Kang and coworkers identified this protein as a key regulator of cellular senescence [53, 130]. GATA4 is also important for DDR and is regulated by autophagy, so it can be at the crossroad of these three cellular phenomena: senescence, autophagy, and DDR (Figure 6). It is also important that GATA4 is involved in the mechanisms inducing SASP phenotype, which can promote chronic inflammation associated with most age-related diseases, including AMD [5, 53, 131]. GATA4 is normally degraded by p62-mediated autophagy and it was shown to be stabilized in cells undergoing senescence, possibly due to a decreased association with p62 [53]. As GATA4 activation depends on the key DDR signaling proteins, ATM and ATR and it is accumulated in some aging tissues, it is a good candidate to orchestrate interplay between senescence, autophagy, and DDR. Kang and coworkers postulated that the GATA4-mediated relationship between autophagy and senescence is different for different modes of autophagy [53]. GATA4 can be a positive regulator of senescence and selective autophagy, but a signal inducing senescence can stimulate GATA4 to avoid selective autophagy.

In searching for a mechanism by which GATA4 regulates senescence, Kang et al. observed that it upregulated (mostly) and downregulated genes important for senescence [53]. Therefore, GATA4 could be involved in the stimulation of a considerable fraction of genes, including the SASP genes, whose expression determines the senescence phenotype. GATA4 was shown to act upstream of NF-*κ*B during senescence induction and depletion of RelA (p65), a component of NF-*κ*B [53]. This effect was associated with the repression of almost all genes involved in SASP, except IL1A. Subsequent experiments showed that GATA4 regulated senescence response independently of the p53 and p16INK4a/Rb pathways.

When key DDR regulators ATM and ATR were inhibited, the GATA4 pathway was inhibited during senescence, suggesting that it is a new independent branch of DDR. The role of DDR in inhibition of autophagy-mediated degradation of GATA4 is not known yet. GATA4 was observed to accumulate in organs of 22-month-old mice as compared to their 6-month-old counterparts, which correlates with the accumulation of senescent cells in aging organism [26, 53, 132–134].

In summary, GATA4 can be involved in DDR and this involvement is independent of p53 and p16^INK4a^/Rb pathways. GATA4 is closely associated with senescence and SASP and is controlled by selective autophagy, but can also stimulate general autophagy [53]. Therefore, it is justified to consider a role of GATA4 in coordinating senescence, autophagy, and DDR. In addition, as GATA4 associates with inflammation, studies on its role in AMD pathogenesis are justified.

## 5. Senescence-Based Pathogenesis of AMD with the Contribution of Autophagy and DDR

RPE cells in the central retina are quiescent due to spatial constraints and contact with neuroretina, and when damaged, they can be replaced by their proliferating counterparts at RPE periphery in an endogenous compensatory mechanism [135]. This endogenous regenerative mechanism is activated in pathological conditions, which can increase with age [136]. Oxidative stress can induce senescence in RPE cells if they are prone to and result in inability of peripheral RPE cells to rescue their central RPE counterparts, which can lead to a massive loss of RPE cells observed in clinically detected AMD. If most of macular peripheral RPE cells are affected by senescence, this mechanism can fail leading to AMD. Senescent RPE will be the source of pathology and have a detrimental impact on surrounding tissue through SASP.

**Figure 7:**
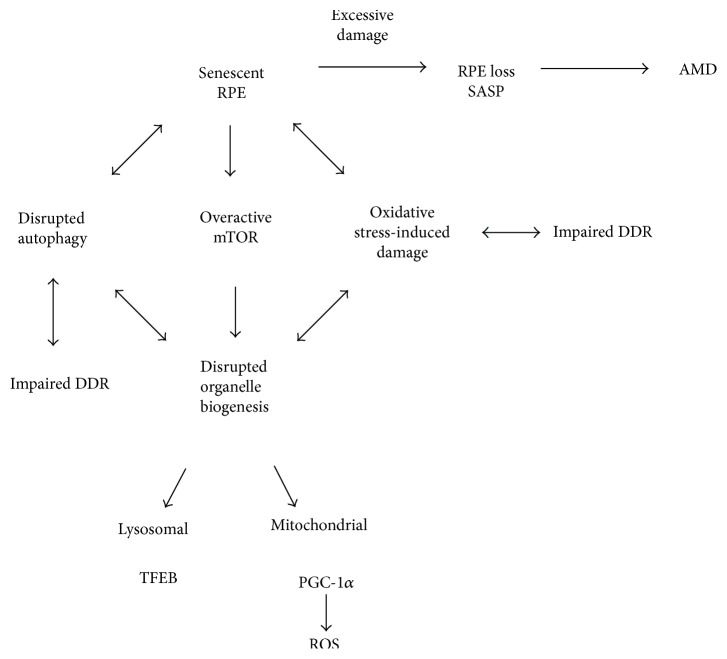
Senescence as a critical factor in AMD pathogenesis. In certain stress conditions, which can be induced by environmental or/and lifestyle factors in aging retina, a major fraction of RPE cells become senescent and are no longer able to regenerate damaged RPE cells, which leads to AMD. The senescence of RPE cells can result from an interplay between aging, autophagy, and DDR in stress conditions. This interplay is a kind of vicious cycle as impaired DNA damage response (DDR) can lead to an increased damage to biomolecules by ROS. Damage to biomolecules induces the degradation of organelles via mTOR-dependent autophagy. This may lead to aggravation of oxidative stress and cellular damage as well as continue to impair autophagy and antioxidant defense by altered TFEB (transcription factor E-box binding) and PGC-1*α* signaling and increased ROS generation.

We hypothesize that under some circumstances, RPE senescence may contribute to or/and precede irreversible pathological events in the retina specific for AMD, such as RPE loss and inflammation. Senescent RPE cells may be excessively damaged, dysfunctional, and capable of overexpression of SASP.  Figure 7 illustrates this novel concept of RPE senescence as a critical contributor to AMD induction and progression.

We believe that senescence associates with autophagy and DDR. As mentioned, cell senescence can be causative for aging and age-dependent diseases. All these three effects, senescence, autophagy, and DDR, can be provoked by oxidative stress, which is a major factor in AMD pathogenesis. Moreover, aging is the main risk factor of pathogenesis of AMD and can be related to oxidative stress [46]. Inflammation associates with oxidative stress, aging (inflammaging), and AMD [5, 137, 138]. Therefore, it is logical and justified to hypothesize that senescence can play a role in AMD and this process can be influenced or regulated by autophagy and DDR. Consequently, GATA4, as an identified factor to be involved in cell senescence, autophagy, DDR, and inflammation, seems to be a natural candidate to play a major role in the proposed mechanism of AMD pathogenesis. However, this is only a hypothesis, which should be verified, but we tried to show some arguments that this subject is worth further study and development.

## 6. Conclusions and Perspectives

Molecular studies on AMD pathogenesis in humans are limited. Therefore, choosing an optimal experimental model for these studies is essential. ARPE-19 cell line is commonly used in molecular research on AMD, even though it is a heterogeneous cell population, including dividing and nondividing cells. However, as AMD is an age-related disease, the process of cell senescence should be included in *in vitro* models. As oxidative stress is a main AMD pathogenesis factor, cellular antioxidant defense is important in the disease prevention. That is why one can use RPE cells from mice knockout of genes essential for antioxidant defense. To include aging in that study, animals at different ages can be used. As the main AMD genetic risk factor are mutations in the gene encoding complement factor H, one can use RPE cells derived from induced pluripotent stem cells (iPSCs) obtained from AMD patients having such mutations.

It seems that the relationship between DDR and autophagy in mitochondria can be especially important for AMD pathogenesis due to many reasons [139]. First, mitochondrial DDR is different from DDR in the nucleus [140]. Second, autophagic mtDNA degradation can be considered as a DDR pathway dealing with heavily damaged molecules of mtDNA [141]. Third, mitochondrial mutagenesis was reported to play a role in AMD pathogenesis [89–91]. Furthermore, the role of mTOR, a crucial autophagy protein, in lysosomal and mitochondrial biogenesis has been recently appreciated [57, 142]. Therefore, studies of DDR and autophagy in mitochondria in the context of AMD are warranted.

To summarize, cellular senescence and SASP can be related to age-related chronic diseases [137, 143]; chronic inflammation (inflammaging) is also involved in age-related chronic diseases [137]; autophagy and senescence seem to be closely related [144]; and lastly the GATA4 protein can be involved in DDR, senescence, and autophagy as well as in inflammation and aging [53, 130].

Work to determine the relationship between aging (senescence), autophagy, and DDR and relate it to AMD can bring information important for AMD clinic and basic molecular biology as there are many essential unanswered questions and problems concerning mutual relationships between aging, autophagy, and cellular reaction to DNA damage.
